# Is Acupuncture Another Good Choice for Physicians in the Treatment of Chronic Prostatitis/Chronic Pelvic Pain Syndrome? Review of the Latest Literature

**DOI:** 10.1155/2020/5921038

**Published:** 2020-03-09

**Authors:** Junjun Li, Liang Dong, Xuhong Yan, Xiaozhang Liu, Ying Li, Xujun Yu, Degui Chang

**Affiliations:** ^1^Hospital of Chengdu University of Traditional Chinese Medicine, Chengdu, China; ^2^The Reproductive & Women-Children Hospital, Chengdu University of Traditional Chinese Medicine, Chengdu, China

## Abstract

This study aimed to evaluate the efficacy and safety of acupuncture for chronic prostatitis/chronic pelvic pain syndrome (CP/CPPS). A search of PUBMED, EMBASE, Central Register of Controlled Trials (CENTRAL), Web of Science, Chinese Biomedicine Literature (CBM), China National Knowledge Infrastructure (CNKI), Wang-Fang Database, Chinese Scientific Journal Database (VIP), and other available resources was made for studies (up to February 2019). Searches were limited to studies published in English and Chinese. Only randomized controlled trials (RCTs) related to the efficacy and/or safety of acupuncture for CP/CPPS were included. Two investigators independently evaluated the quality of the studies. A total of 11 studies were included, involving 748 participants. The results revealed that compared with sham acupuncture (MD: −6.53 [95% CI: −8.08 to −4.97]) and medication (MD: −4.72 [95% CI: −7.87 to −1.56]), acupuncture could lower total NIH-CPSI score more effectively. However, there are no significant differences between acupuncture and sham acupuncture in terms of IPSS score. In terms of NIH-CPSI voiding domain subscore, no significant differences were found between acupuncture and medication. Compared with sham acupuncture (OR: 0.12 [95% CI: 0.04 to 0.40) and medication (OR: 3.71 [95% CI: 1.83 to 7.55]), the results showed favorable effects of acupuncture in improving the response rate. Acupuncture plus medication is better than the same medication in improving NIH-CPSI total score and NIH-CPSI pain domain subscore. In conclusion, the evidence suggests that acupuncture may be an effective intervention for patients with CP/CPPS. However, due to the heterogeneity of the methods and high risk of bias, we cannot draw definitive conclusions about the entity of the acupuncture's effect on alleviating the symptoms of CP/CPPS. The adverse events of acupuncture are mild and rare.

## 1. Introduction

Chronic prostatitis/chronic pelvic pain syndrome (CP/CPPS) is a complex clinical entity consisting of urogenital pain, lower urinary tract symptoms, and/or sexual dysfunction that lasts for at least 3 months in the past 6 months [1]. CP/CPPS exists in more than 90–95% of patients with prostatitis [2], and it is estimated to affect 2–15% of adult men [3–6]. The lifetime prevalence of CP/CPPS is about 2.2% to 8.2% [7], and its main clinical symptoms are summarized as UPOINT, including Urinary symptoms, Psychosocial dysfunction, Organ-specific findings, Infection, Neurological dysfunction, and Tenderness of muscles. These symptoms, especially chronic pelvic pain syndrome, persist for a long time and are difficult to recover, which seriously affects the quality of life of patients.

CP/CPPS is a severe challenge and difficult problem for urologists, and there is no “golden standard” to treat the disease because pathogenesis remains unclear and some researchers think CP/CPPS is a multifactorial disease such as abnormal immune response, genetic predisposition, pathogen infection, neuromuscular factors, and intraprostatic ductal reflux [8]. So it is essential to find an appropriate treatment for CP/CPPS.

At present, antibiotics, anti-inflammatory drugs, alpha-blockers, and neuromodulators are the most commonly used drugs in the treatment of CPPS. However, the use of antibiotics remains controversial because there are no isolated bacteria [9]. Antiobstructive drugs that reduce pain and alpha-blockers that improve outflow tract obstruction should be taken within a limited period of time to offset the side effects [10]. Hence, more and more attention has been paid to phytotherapy and physiotherapy with less adverse reactions and high acceptance of patients in recent years.

Acupuncture is a form of alternative medicine and a key component of traditional Chinese medicine (TCM). It is most often used to relieve pain, though it is also recommended by acupuncturists for a wide range of other conditions. Different methods are used during acupuncture such as manual manipulation, electrical stimulation, and heat. Another form of acupuncture is acupoint catgut embedding. Studies reported that acupuncture had the effect of anti-inflammatory, immune modulation and neuromodulation. In 2016, Liu et al. [11] conducted a systematic review that shows acupuncture is effective in the treatment of CP/CPPS. It can relieve pain symptoms, reduce National Institute of Health Chronic Prostatitis Symptom Index (NIH-CPSI) scores, and improve the quality of life of patients with CP/CPPS. However, due to insufficient number of high-quality, well-designed, randomized controlled trials (RCTs), the effect of acupuncture on CP/CPPS is limited [12]. In the past three years, some new RCTs have been published on CP/CPPS treating with acupuncture. Therefore, a comprehensive and systematic evaluation should be carried out.

## 2. Materials and Methods

The style of reporting the findings in the manuscript was performed in accordance with the Preferred Reporting Items for Systematic Review and Meta-Analysis (PRISMA) statement [13].

### 2.1. Search Methods

A search of PUBMED, EMBASE, CENTRAL, Web of Science, CBM, CNKI, Wang-Fang Database, VIP, and other available resources was made for studies (up to February 2019) that compared the efficacy and safety of acupuncture with sham acupuncture or medication (such as alpha-adrenergic antagonist, antibiotics, or anti-inflammatory drugs). The search terms related to acupuncture, chronic prostatitis, chronic pelvic pain syndrome, and randomized controlled trials. Searches were limited to studies published in English and Chinese (see Table 1 for PUBMED database search strategy).

### 2.2. Eligibility Criteria

#### 2.2.1. Types of Studies

Only RCTs related to the efficacy and/or safety of acupuncture for CP/CPPS were included. Trials used in meta-analysis should include the descriptions of adequate randomization methods, qualification diagnosis, qualification results reporting, and statistical methods. The quality of studies was evaluated by professional assessors. Articles focused on mechanisms, expert experience, animal experiments, reviews, and those without full text were excluded.

#### 2.2.2. Types of Participants

Participants diagnosed with CP/CPPS (category III as classified by the NIH) were included. CP/CPPS was defined as urogenital pain, lower urinary tract symptoms, and/or sexual dysfunction that lasts for at least 3 months in the past 6 months in the absence of any urinary tract infection. Participants with benign prostatic hypertrophy, acute bacterial prostatitis, prostate cancer, severe heart disease, hepatic and kidney dysfunction, severe mental disease, or other serious diseases were excluded.

#### 2.2.3. Types of Intervention

Acupuncture compared to western drugs, acupuncture with drugs compared to the same drugs, and acupuncture compared to sham acupuncture were included. In addition, for the purposes of this review, we focused on acupuncture that can be performed in primary care settings, including any type of penetrating acupuncture (i.e., acupuncture, electroacupuncture, warm acupuncture, abdominal acupuncture, auricular acupuncture, acupoint catgut embedding, etc.). Comparison of two different types of acupuncture, acupuncture injections and acupuncture combined/compared with Chinese herbal medicine or acupuncture as a supplement to the effectiveness of the above interventions was excluded.

#### 2.2.4. Types of Outcome Measures

Changes in the total NIH-CPSI score [14], NIH-CPSI subscales, International Prostate Symptom Score (IPSS), and global response rate after treatment were recorded. In addition, adverse events from interventions were also recorded.

### 2.3. Data Collection

We extracted the information of characteristics of participants, types of treatments and control groups, outcome measures, adverse events, and the follow-up period, if available (see Tables 2 and 3). For the purpose of this review, we extracted the change score of means and standard deviation, and when the data in the test report is insufficient, we try to contact the author. We estimated data using the methods recommended in the Cochrane Handbook for Systematic Reviews of Interventions if no one responded [26].

### 2.4. Data Synthesis and Analysis

The measurement scales used to evaluate therapeutic effects were the NIH-CPSI (three domains: pain, voiding, and QoL; scores 0–43) and the IPSS (two domains: voiding and storage, scores 0–35). The scores of the acupuncture and control groups at the end of the original study period were compared. Response rate was defined according to the definitions in the original studies.

All analyses were performed by the Review Manager statistical software (version 5.3). The continuous outcomes were analyzed using mean difference (MD) as the summary statistic. The dichotomous outcomes were analyzed using odds ratios (ORs) as the summary statistic. *X*^2^ statistical tests (*Q* statistics) and the *I*^2^ test were used to test the heterogeneity between the trials. The parameters with mean value and 95% confidence interval were transformed into mean values with standard deviation for calculation of weighted mean difference.

### 2.5. Risk of Bias Assessment

Cochrane Collaboration tool [27]was used to evaluate the risk of bias for the RCTs included. Two investigators (JL and LD) independently evaluated the quality of the studies. References of previous published meta-analysis that met the enrollment criteria were included for pooled analysis. When discrepancies occurred, a third investigator (JY) is consulted to reach a consensus.

## 3. Results

**Figure 1:**
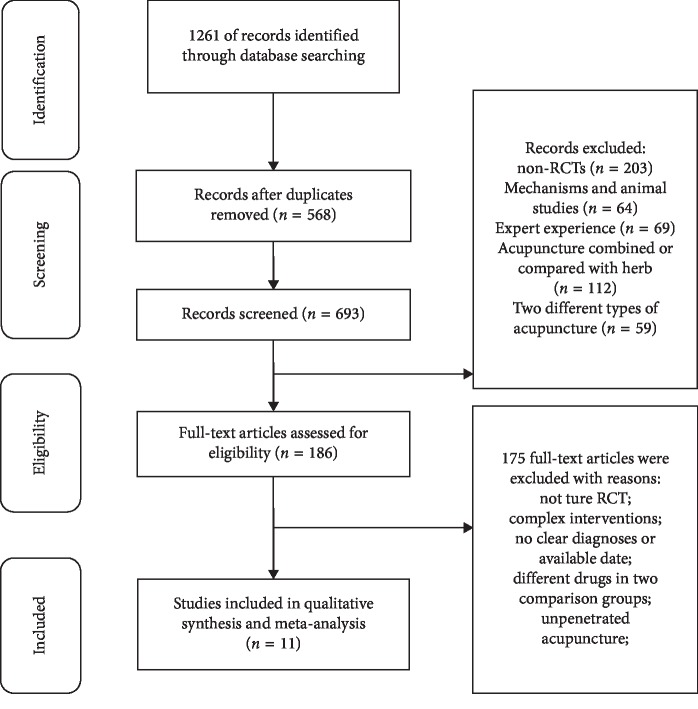
Flow chart for the selection of trials.

As shown in the flow chart of selection (Figure 1), a total of 1261 studies were searched, but only 693 studies were included for evaluation after duplications were removed. After excluding abstracts irrelevant to the topic, the full texts of 186 studies were retrieved for evaluation. Studies with inappropriate interventions, participants with bacteria prostatitis, or other prostate diseases were excluded. Studies without clear diagnosis and available date were also excluded.

Finally, a total of 11 studies [15–25] were included for quantity and quality analysis in this review. Five trials published in English were from Malaysia [15], Korea [16], Turkey [21, 22], and China [25], and the remaining 6 [17–20, 23, 24] were all from China and published in Chinese. All 11 trials were single centre, RCTs. The interventions included 2 trials of electroacupuncture (EA) [16, 21], 1 trial of catgut embedding therapy [19], and 8 trials of manual acupuncture (MA) [15, 17, 18, 20, 22–25], in which 2 trials used MA plus medicine [17, 23] (see Table 2). In the control group, sham acupuncture included selection of nonacupoints (superficial and/or 10–15 mm to the left of each correct acupoint) and medication included Tamsulosin Hydrochloride, Prostate Tablets, Terazosin, indomethacin, ibuprofen, and levofloxacin.

**Figure 2:**
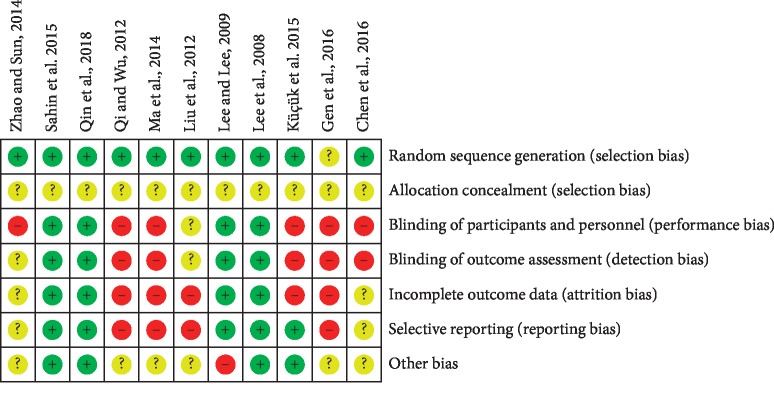
ROB for included trials.+, low risk of bias; ?, unclear; −, high risk of bias.

The quality of the enrolled studies was evaluated by the Cochrane Collaboration tool. Information of all included RCTs on subsequent allocation is not clear, thus all 11 trials were rated as having unclear risk of bias in this domain. Six RCTs comparing acupuncture to medication did not provide sufficient blinding information [17, 19–21, 23, 24], and we believe that the limitations of this approach may affect the results. Therefore, those 6 trials are considered to have a high risk of bias in blinding domain. Four trials provided a total NIH-CPSI score without subscores and no adverse events or dropoffs, we considered a high risk of incomplete data and selective outcome reporting [17–19, 24]. One RCT of a small group size was rated as high risk in other biased domains [16]. One RCT only said the trial was random but did not explain the random method; the trial was rated as having unclear risk of bias in random sequence generation domain [24](Figure 2).

### 3.1. Acupuncture versus Sham Acupuncture

**Figure 3:**
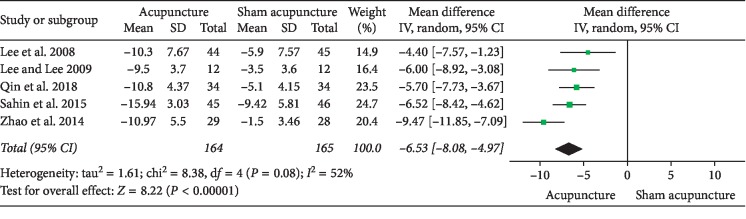
Forest plot of comparisons of NIH-CPSI total score (acupuncture vs. sham acupuncture).

**Figure 4:**
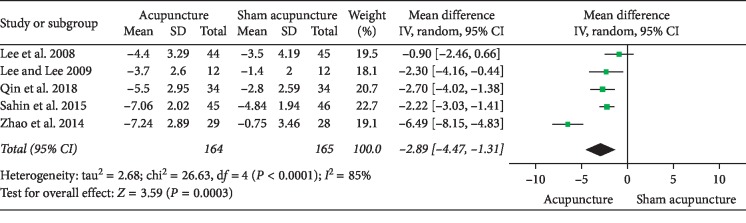
Forest plot of comparisons of NIH-CPSI pain domain subscore (acupuncture vs. sham acupuncture).

**Figure 5:**
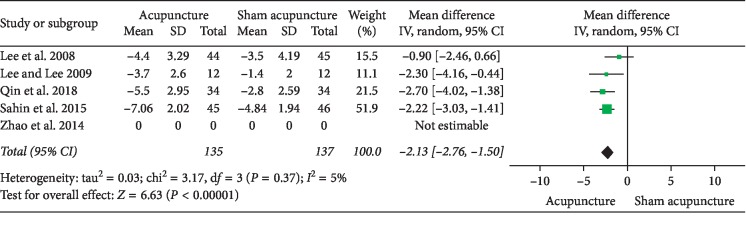
Forest plot of comparisons of NIH-CPSI pain domain subscores after eliminating Zhao 2014 (acupuncture vs. sham acupuncture).

**Figure 6:**
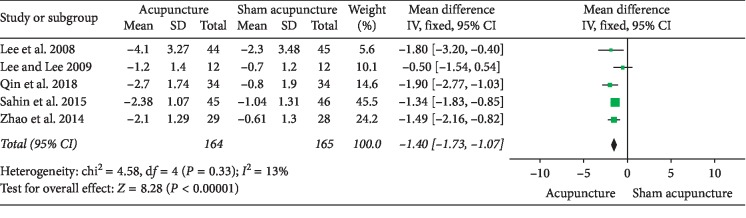
Forest plot of comparisons of NIH-CPSI voiding domain subscore (acupuncture vs. sham acupuncture).

**Figure 7:**
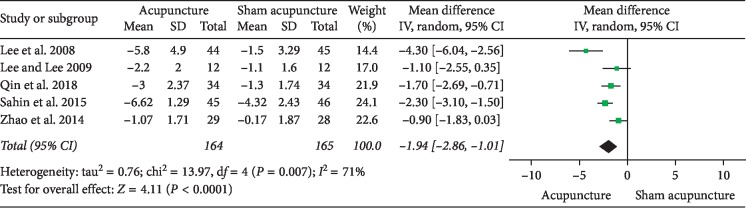
Forest plot of comparisons of NIH-CPSI quality of life domain subscore (acupuncture vs. sham acupuncture).

**Figure 8:**
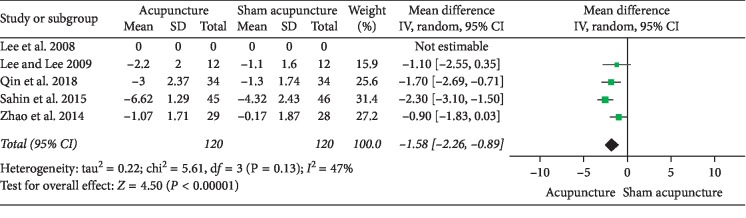
Forest plot of comparisons of NIH-CPSI quality of life domain subscore. After eliminating Lee, 2008 (acupuncture vs. sham acupuncture).

**Figure 9:**
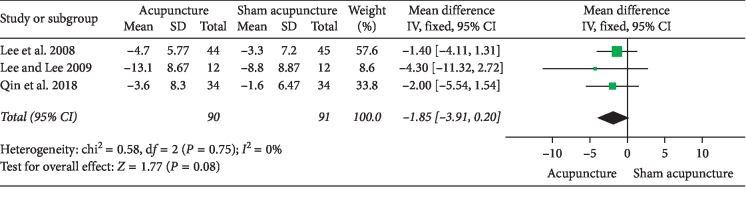
Forest plot of comparisons of IPSS (acupuncture vs. sham acupuncture).

**Figure 10:**
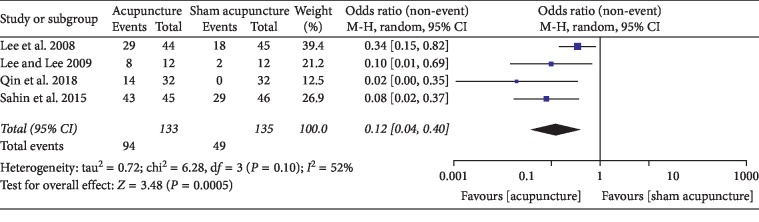
Forest plot of comparisons of response rate (acupuncture vs. sham acupuncture).

- NIH-CPSI total score: 5 RCTs involving 329 participants evaluated total NIH-CPSI total score as an outcome. Meta-analysis showed that acupuncture yielded a significant decrease in the total NIH-CPSI score (MD: −6.53 [95% CI: −8.08 to −4.97]) with moderate heterogeneity (*I*^2^ = 52%) (Figure 3).
- NIH-CPSI pain domain subscore: in the pain domain score, 5 RCTs involving 329 participants were included in a meta-analysis. The results showed an average pain score reduction of 2.89 points (MD: −2.89 [95% CI: −4.47 to −1.31]) with high heterogeneity (*I*^2^ = 85%) (Figure 4). A sensitivity analysis succeeded in identifying the source of heterogeneity: 1 trial conducted by Zhao. After eliminating the trail from the data combination, the heterogeneity decreased significantly and could be accepted (*I*^2^ = 5%) with an average pain reduction of 2.13 (MD: −2.13 [95% CI: 2.76 to −1.50]) (Figure 5).
- NIH-CPSI voiding domain subscore: 5 RCTs involving 329 participants that compared acupuncture to sham acupuncture reported changes in the NIH-CPSI voiding domain subscore. Meta-analysis showed a significant improvement in acupuncture compared to sham acupuncture (MD: −1.40 [95% CI: −1.73 to −1.07]) with low heterogeneity (*I*^2^ = 13%) (Figure 6).
- NIH-CPSI quality of life domain subscore: in terms of improving quality of life, a meta-analysis of 5 trials involving 329 participants showed that acupuncture can improve the quality of life of patients with CP/CPPS compared with sham acupuncture (MD: −1.94 [95% CI: −2.86 to −1.01]) with high heterogeneity (*I*^2^ = 71%) (Figure 7). After sensitivity analysis, we eliminated trial of Lee 2008; the heterogeneity decreased and could be accepted (*I*^2^ = 47%) with an average pain reduction of 1.58 (MD: −1.58 [95% CI: −2.26 to −0.89]) (Figure 8).
- IPSS score: 3 trials involving 181 participants evaluated total IPSS score as a secondary outcome. In the results of meta-analysis, no significant differences were found between acupuncture and sham acupuncture (MD: −1.85 [95% CI: −3.91 to 0.20]) with *I*^2^ = 0% (Figure 9).
- Response rate: 4 RCTs involving 268 participants that compared acupuncture to sham acupuncture reported response rate. According to these trials, a participant who has a decrease more than 4 to 6 points in total NIH-CPSI score after treatment can be considered a responder. A meta-analysis of the data showed favorable effects of acupuncture on improving the response rate (OR: 0.12 [95% CI: 0.04 to 0.40]) with moderate heterogeneity (*I*^2^ = 52%) (Figure 10).

### 3.2. Acupuncture versus Medication

**Figure 11:**
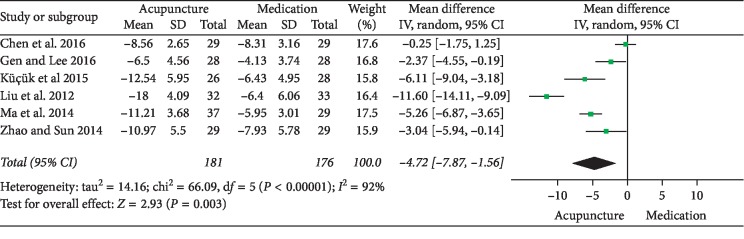
Forest plot of comparisons of NIH-CPSI total score (acupuncture vs. medication).

**Figure 12:**
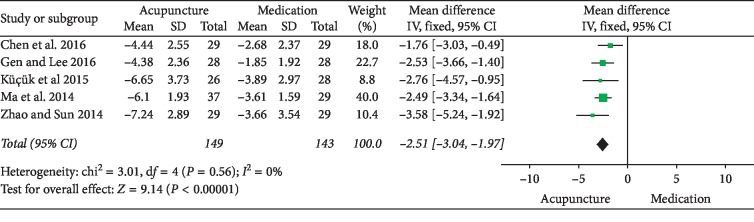
Forest plot of comparisons of NIH-CPSI pain domain subscore (acupuncture vs. medication).

**Figure 13:**
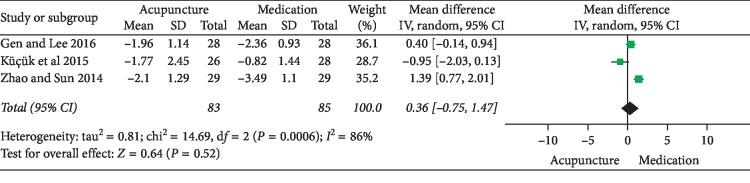
Forest plot of comparisons of NIH-CPSI voiding domain subscore (acupuncture vs. medication).

**Figure 14:**
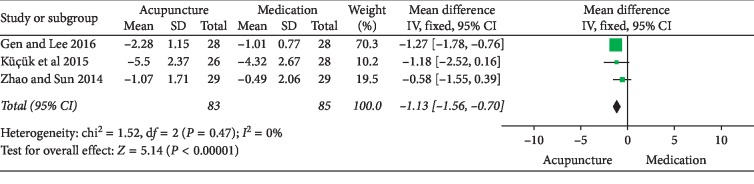
Forest plot of comparisons of NIH-CPSI quality of life domain subscore (acupuncture vs. medication).

**Figure 15:**
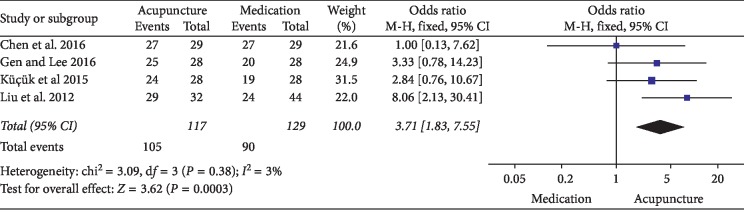
Forest plot of comparisons of response rate (acupuncture vs. medication).

- NIH-CPSI total score: 6 RCTs involving 357 participants comparing acupuncture to medication reported changes in the total NIH-CPSI score. Meta-analysis of 6 trials yielded a significant difference in favor of acupuncture (MD: −4.72 [95% CI: −7.87 to −1.56]) with high heterogeneity (*I*^2^ = 92%) (Figure 11). Because of the insufficient studies included, subgroup analyses or sensitivity analyses failed to explore the source of heterogeneity. As a result, the evidence of combing data has been limited. The source of heterogeneity may relate to different acupoints selected.
- NIH-CPSI pain domain subscore: 5 RCTs involving 292 participants compared acupuncture to medication. In the pain domain score, meta-analysis showed an average pain score reduction of 2.51 points (MD: −2.51 [95% CI: −3.04 to −1.97]) with low heterogeneity (*I*^2^ = 0%) (Figure 12).
- NIH-CPSI voiding domain subscore: 3 RCTs involving 168 participants that compared acupuncture to medication in NIH-CPSI voiding domain subscore. The results showed that there was no significant difference between the acupuncture and medication (MD: 0.36 [95% CI: −0.75 to 1.47]) with high heterogeneity (*I*^2^ = 86%) (Figure 13). Because of the insufficient studies included, subgroup analyses or sensitivity analyses failed to explore the source of heterogeneity.
- NIH-CPSI quality of life domain subscore: for improvement in quality of life, the result of meta-analysis of 3 trials involving 168 participants indicated that compared with medication, acupuncture could improve the quality of life in patients with CP/CPPS better (MD: −1.13 [95% CI: −1.56 to −0.70]) with low heterogeneity (*I*^2^ = 0%) (Figure 14).
- Response rate: 4 trials involving 246 participants reported global assessment as one of the outcomes. A meta-analysis of the data showed favorable effects of acupuncture in improving the response rate (OR: 3.71 [95% CI: 1.83to 7.55]) with low heterogeneity (*I*^2^ = 3%) (Figure 15).

### 3.3. Acupuncture plus Medication versus the Same Medication

**Figure 16:**
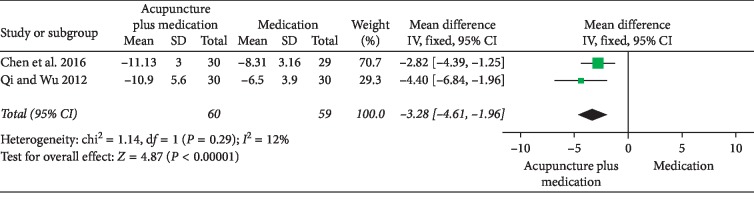
Forest plot of comparisons of NIH-CPSI total score (acupuncture plus medication vs. medication).

**Figure 17:**
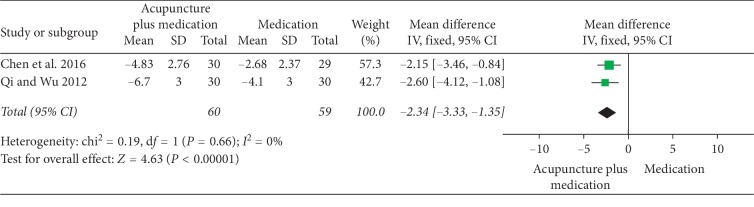
Forest plot of comparisons of NIH-CPSI pain domain subscore (acupuncture plus medication vs. medication).

- NIH-CPSI total score: 2 RCTs involving 119 participants comparing acupuncture plus medication to the same medication reported changes in the total NIH-CPSI score. Meta-analysis of 2 trials yielded a significant difference in favor of acupuncture plus medication (MD: −3.28 [95% CI: −4.61 to −1.96]) with low heterogeneity (*I*^2^ = 12%) (Figure 16).
- NIH-CPSI pain domain subscore: 2 RCTs involving 119 participants compared acupuncture plus medication to the same medication reported changes in the NIH-CPSI pain domain subscore. Meta-analysis of the data showed favorable effects of acupuncture plus medication (MD: −2.34 [95% CI: −3.33 to −1.35]) with low heterogeneity (*I*^2^ = 0%) (Figure 17).

### 3.4. Adverse Events

Four of the 11 trials reported the occurrence of adverse events (ADs) in the acupuncture group [15, 16, 20, 25], 2 trials reported no ADs [21, 22], and the rest did not provide information related to ADs [17–19, 23, 24] (see Table 3 for details).

## 4. Discussion

This study is a systematic review and meta-analysis of the therapeutic effect of acupuncture on patients with CP/CPPS. To investigate the efficacy of acupuncture, we combined the experimental data to calculate the mean difference by comparing the baseline and endpoint results of the control group. The results show that acupuncture is superior to sham acupuncture in terms of NIH-CPSI total score (MD: −6.53 [95% CI: −8.08 to −4.97], *P* < 0.05), NIH-CPSI pain domain subscore (MD: −2.89 [95% CI: −4.47 to −1.31], *P* < 0.05), NIH-CPSI voiding domain subscore (MD: −1.40 [95% CI: −1.73 to −1.07], *P* < 0.05), NIH-CPSI quality of life domain subscore (MD: −1.94 [95% CI: −2.86 to −1.01], *P* < 0.05), and response rate (OR: 0.12 [95% CI: 0.04 to 0.40], *P* < 0.05). However, there is no significant difference between acupuncture and sham acupuncture in improving IPSS (MD: −1.85 [95% CI: −3.91 to 0.20], *P*=0.08), which is different from previous meta-analyses results [28, 29], which demonstrated that acupuncture can improve IPSS better than sham acupuncture. The contradiction of sources is difficult to determine because subgroup analysis and sensitivity analysis are not possible without the availability of additional information from other trials. Compared to medication, the pooled results reveal that acupuncture is superior to this standard drug therapy as regards NIH-CPSI total score (MD: −4.72 [95% CI: −7.87 to −1.56], *P* < 0.05), NIH-CPSI pain domain subscore (MD: −2.51 [95% CI: −3.04 to −1.97], *P* < 0.05), NIH-CPSI quality of life domain subscore (MD: −1.13 [95% CI: −1.56 to −0.70], *P* < 0.05), and response rate (OR: 3.71 [95% CI: 1.83 to 7.55], *P* < 0.05), except NIH-CPSI voiding domain subscore (MD: 0.36 [95% CI: −0.75 to 1.47], *P* > 0.05). In addition, this review reveals that acupuncture plus medication is better than the same medication in improving NIH-CPSI total score (MD: −3.28 [95% CI: −4.61 to −1.96], *P* < 0.05) and NIH-CPSI pain domain subscore (MD: −2.34 [95% CI: −3.33 to −1.35], *P* < 0.05). As a result, acupuncture may be recommended as a major treatment for patients with CP/CPPS who have no signs of bacterial infection. However, the small number of cases and the variety of treatment options hinder the determination of the efficacy of acupuncture treatment. More high-quality RCTs are needed to verify the exact efficacy of acupuncture for CP/CPPS.

There are different theories regarding the aetiology and pathophysiology of CP/CPPS, including infection [30–32], inflammation/autoimmunity [33], neuropsychological factors [34, 35], adrenal axis abnormalities [36], pelvic floor muscles dysfunction [37, 38], pelvic nerve entrapment [38], genetic predisposition to inflammation [39] and oxidative stress [40]. Due to the diversity of aetiology and pathophysiology of CPPS, standard drug treatment is unsatisfactory. The efficacy of antibiotics, alpha-blockers, and anti-inflammatory drugs has been reported to be variable and frustrating. NIH-funded studies show that the efficacy of drug treatment for CPPS is negative [41, 42]. Phytotherapy, such as low-energy shock wave, has also been reported in recent years for pelvic floor diseases, such as CP/CPPS [43] and erectile dysfunction [44]. Therefore, many alternative therapies have been proposed, including phytotherapy, lifestyle changes, physical therapy, diet, cognitive behavioral therapy, and acupuncture [45].

Acupuncture, which is one of the most commonly used nondrug therapies, has been used to treat symptoms of CP/CPPS patients in many countries. According to a report published by the World Health Organization [46], acupuncture has been widely used in various physiological diseases, including pain, infection, nervous system diseases, and urogenital diseases. However, the mechanism of the role of acupuncture is still unclear. At present, acupuncture is regarded as sensory nerve stimulus [47, 48]. It has been used to relieve pain based on evidence of biological mechanisms and has been widely used in chronic diseases such as myofascial pain, muscle diseases, and neurological diseases in eastern countries [49]. The analgesic effect of acupuncture on CP/CPPS may involve levels of events on the tissue, spinal cord, and supraspinal, including regulation of the endogenous opioid system, gate control therapy, and the purinergic signaling system. In addition, increasing the levels of endomorphin-1, beta-endorphin, encephalin, and serotonin may also be involved [50]. Acupuncture may also improve CP/CPPS symptoms by modulating the activity of immune cells and the secretion of immune molecules. Lee et al. [51] showed that acupuncture could increase the ratio of CD3+, CD4+, CD8+, CD19+, and NK cells, indicating that acupuncture can alleviate symptoms by modulating the immune system of CP/CPPS.

In this study, 5 trials did not provide information related to ADs [17–19, 23, 24]. Two trials reported no ADs [21, 22]. Hematoma and pain at the needle site were reported in both the acupuncture and sham acupuncture groups in Lee 2008's finding [15]. Lower back pain near the needling was reported in sham electroacupuncture group in Lee 2009's study [16]. Qin, in 2018, revealed that hematoma occurred in 3 participants and 1 participant described sharp needling pain in acupuncture group; fatigue occurred in 1 participant in sham acupuncture group [25]. Zhao and Sun 2014 reported that 1 participant fainted during acupuncture treatment and 1 participant had hypotension after taking Tamsulosin [20]. Most studies reported little side effects associated with acupuncture. Acupuncture is, hence, a safe treatment for CPPS. Unskillful with acupuncture is an important factor in the occurrence of acupuncture side effects.

This article has several limitations. First, all trials lack details of concealment, and most of them do not provide enough information about blind methods. Due to the characteristics of acupuncture, it is difficult for patients to be treated blindly, especially in case of using drugs in the control group. Second, the reaction time of acupuncture may be problematic because most studies have short-term follow-up, and there is very little data on the effects of repeated acupuncture. Third, there are still few high-quality studies comparing acupuncture with standard drug therapy. The small sample size of the study included may lead to publication bias. Fourth, different types of acupuncture, frequency of treatment, duration, and location of each course of treatment may have a potential impact on acupuncture. Due to insufficient trials, subgroup analysis or metaregression is difficult to avoid the limitations of this methodology. At last, this study did not determine which patients might benefit from acupuncture and which stimuli (pure needle, electrical, or catgut embedding) performed better. Although the current meta-analysis shows encouraging results, further research is necessary to determine what kinds of patients could benefit from acupuncture.

## 5. Conclusion

Acupuncture may be an effective intervention for patients with CP/CPPS. Compared with sham acupuncture, real acupuncture yielded a significant reduction in the NIH-CPSI score. Compared with medication, acupuncture is better in improving NIH-CPSI total score, pain domain subscore, and quality of life domain subscore. In addition, acupuncture plus medication is better than the same medication in improving NIH-CPSI total score and NIH-CPSI pain domain subscore. However, due to the heterogeneity of the methods and high risk of bias, we cannot draw definitive conclusions about the entity of the acupuncture's effect on alleviating the symptoms of CP/CPPS. The adverse events of acupuncture are mainly hematoma and local pain, which could be quickly relieved, and no other serious side effects were found.

## Figures and Tables

**Table 1:** PUBMED database search strategy.

((Acupuncture[MeSH] OR Acupuncture[Title/Abstract] OR Pharmacopuncture[Title/Abstract]) OR Acupuncture Therapy[MeSH] OR Acupotom^*∗*^ [Title/Abstract] OR (Auricular[Title/Abstract] OR Ear[Title/Abstract]) OR (Electroacupuncture[Title/Abstract])) AND (Prostatitis[MeSH] OR Prostatiti^*∗*^[Title/Abstract] OR Chronic pelvic pain syndrome[Title/Abstract] OR Non-bacterial prostatitis[Title/Abstract]) AND ((randomize controlled trial[pt] OR controlled clinical trial[pt] OR randomized[Title/Abstract] OR placebo[Title/Abstract] OR clinical trials as topic[MeSH:noexp] OR randomly[Title/Abstract] OR trial[Title/Abstract]) NOT (animals[MeSH] NOT humans[MeSH]))

**Table 2:** The methods of acupuncture and chosen acupoints of the enrolled studies.

References	Acupuncture and acupoints
Lee et al., 2008 [15]	Needle acupuncture, 4 points; CV1 (GuanYuan), CV4 (Huiyin), SP6 (Saninjiao), SP9 (Yinlinquan)
Lee and Lee, 2009 [16]	Electroacupuncture, 6 points; bilaterally; BL32 (zhongliao), BL33 (ciliao), GB30 (huantiao)
Qi and Wu, 2012 [17]	Needle acupuncture, 7 points; CV1 (Huiyin), CV3 (Zhongji), CV4 (GuanYuan), SP9 (Yinlingquan, bilateral), SP10 (Xuehai, bilateral)
Liu et al., 2012 [18]	Needle acupuncture, 6 points; CV4 (GuanYuan), ST28 (Shuidao), SP6 (Sanyinjiao), LIV3 (Tai Chong), EXHN1 (Sishencong), BL54 (Zhibian)
Ma et al., 2014 [19]	Catgut embedding therapy; SP6 (Sanyınjiao), CV2 (Qugu), CV1 (Huiyin), ST36 (Zusanli), CV3 (Zhongji), BL23 (Shenshu)
Zhao and Sun, 2014 [20]	Needle acupuncture, 3 points; LU7 (Lieque), SI3 (Houxi), SP4 (Gongshun)
Küçük et al., 2015 [21]	Electroacupuncture, 6 points; UB 28 (Pang Guang Shu), GB 41 (Zu Lin Qi), LIV 3 (Tai Chong), SP 6 (Sanyinjiao), SP 8 (Diji), LI 4 (He Gu)
Sahin et al., 2015 [22]	Needle acupuncture, 7 points; BL33 (Zhongliao), BL34 (Xialiao), BL54 (Zhibian), CV1 (Huiyin), CV4 (Guanyuan), SP6 (Sanyinjiao), SP9 (Yinlingquan)
Chen et al., 2016 [23]	Needle acupuncture;
	Head-points:GV24 (Shenting), GV22 (Xinhui), GV21 (Qianding), GV20 (Baihui), BL6 (Chengguang), BL7 (Tongtian), etc.
	Body-points:CV3 (Zhongji), BL28 (Pangguangshu), BL32 (Ciliao), etc.
Gen et al. 2016, [24]	Needle acupuncture;
	Head-points:EXHN1 (Sishencong), GV20 (Baihui);
	Abdomen-points:CV3 (Zhongji), CV4 (Guanyuan), CV6 (Qihai);
	Leg-points:SP9 (Yinlingquan), GB34 (Yanglingquan), SP6 (Sanyınjiao), ST36 (Zusanli).
Qin et al. 2018, [25]	Needle acupuncture, 4 points; BL33 (Zhongliao), BL23 (Shenshu), BL35 (Huiyang), SP6 (Sanyinjiao)

**Table 3:** The baseline characteristics of the patients of the enrolled studies.

References	Patient age, years	Inclusion criteria	Control intervention	Sample size (acupuncture vs. control)	Outcomes	Acupuncture sessions	Follow-up time	Adverse events (acupuncture vs. control)
Lee et al., 2008 [15]	40.9 ± 11.0 (Acu) vs. 42.8 ± 9.4 (Sacu)	CP/CPPS	Sham acupuncture	44 (Acu):45 (Sacu)	NIH-CPSI	Biweekly for 10 weeks	5, 10, 14, 22, 34 weeks	8/44 (6 hematomas and 2 with pain at needling sites) vs. 5/45(1 hematoma, 3 with pain at needling sites, and 1 with acute urinary retention)
Lee and Lee, 2009 [16]	39.8 ± 5.8 (Acu) vs. 36.4 ± 5.8 (Sacu)	CP/CPPS (category III)	Sham acupuncture	12 (Acu):12 (Sacu)	IPSS、NIH-CPSI Brief pain inventor	Biweekly for 6 weeks	3, 6 weeks	Only 1 Sacu patient experienced lower back pain near the needling site.
Sahin et al., 2015 [22]	32.1 ± 7.2 (Acu) vs. 32.8 ± 7.0 (Sacu)	CP/CPPS (category III B)	Sham acupuncture	45 (Acu):46 (Sacu)	IPSS NIH-CPSI	Every week for 6 weeks	6, 8, 16, 24 weeks	No adverse events were reported in both groups.
Qin et al., 2018 [25]	33.8 ± 6.8 (Acu) vs. 35.1 ± 9.6 (Sacu)	CP/CPPS	Sham acupuncture	34 (Acu):34 (Sacu)	NIH-CPSI IPSS	3 times a week for 8 weeks	24 weeks	4/34(3 participants complained of hematoma and 1 described sharp needling pain) vs. 1/34 (1 participant reported fatigue after treatment)
Zhao and Sun, 2014 [20]	32 ± 6. 91 vs. (Acu) 33 ± 7. 39 (Sacu) vs. 31 ± 6. 78 (Med)	CP/CPPS (category III B)	Sham acupuncture; Tamsulosin Hydrochloride 0.2 mg qd (Med)	29 (Acu):29 (Sacu):29 (Med)	NIH-CPSI	Biweekly for 4 weeks	No report	1 (Acu, 1 participant fainted during treatment) vs. 0 (Sacu) vs. 1 (Med, 1 participant had hypotension)
Liu et al., 2012 [18]	33.2 ± 10.6 (Acu) vs. 31.8 ± 8.8 (Med)	CP (not specified)	Prostate tablets 70 mg bid,	33 (Acu):32 (Med)	NIH-CPSI	3 times a week for 4 weeks	No report	Not provided
Qi and Wu, 2012 [17]	32.60 ± 7.04 (Acu + Med) vs. 34.77 ± 10.88 (Med)	CP/CPPS (category III)	Terazosin 2 mg qd	30 (Acu + Med):30 (Med)	NIH-CPSI	Once every three days, a total of 10 times	No report	Not provided
Ma et al., 2014 [19]	31 ± 8 (Acu) vs. 33 ± 7.0 (Med)	CP (category III B)	Tamsulosin Hydrochloride 0.2 mg, indomethacin 75 mg tid	37 (Acu):29 (Med)	NIH-CPSI Chinese medicine symptom score	Every 2 weeks for 8 weeks	8 weeks	Not provided
Küçük et al., 2015 [21]	33.30 ± 7.84 (total)	CP/CPPS (category III B)	Levofloxacin 500 mg daily, ibuprofen 200 mg bid	26 (Acu):28 (Med)	NIH-CPSI	Twice a week for 7 weeks	28 weeks (range 20–43 weeks)	No adverse events were reported in both groups.
Chen et al., 2016 [23]	33 ± 7 (Acu) vs. 34 ± 7 (Med)<	CP/CPPS	Levofloxacin 200 mg bid, Tamsulosin Hydrochloride 0.2 mg qd	30 (Acu + Med):29 (Acu):29 (Med)	NIH-CPSI	Once a day for 24 days	No report	Not provided
Gen et al., 2016 [24]	29.13 ± 13.56 (Acu) vs. 28.84 ± 14.63 (Med)	CP/CPPS (category III B)	Tamsulosin Hydrochloride 0.2 mg qd	28 (Acu):28 (Med)	NIH-CPSI	Once every 2 days, for 4 weeks	No report	Not provided

Acu: acupuncture; sacu: sham acupuncture; med: medication.
